# Supplementary figures and images for: Wnt3 distribution in the zebrafish brain is determined by expression, diffusion and multiple molecular interactions
Source: eLife. 2020 Nov 25;9:e59489. doi: 10.7554/eLife.59489 (PMC7725503; doi:10.7554/eLife.59489)

**A**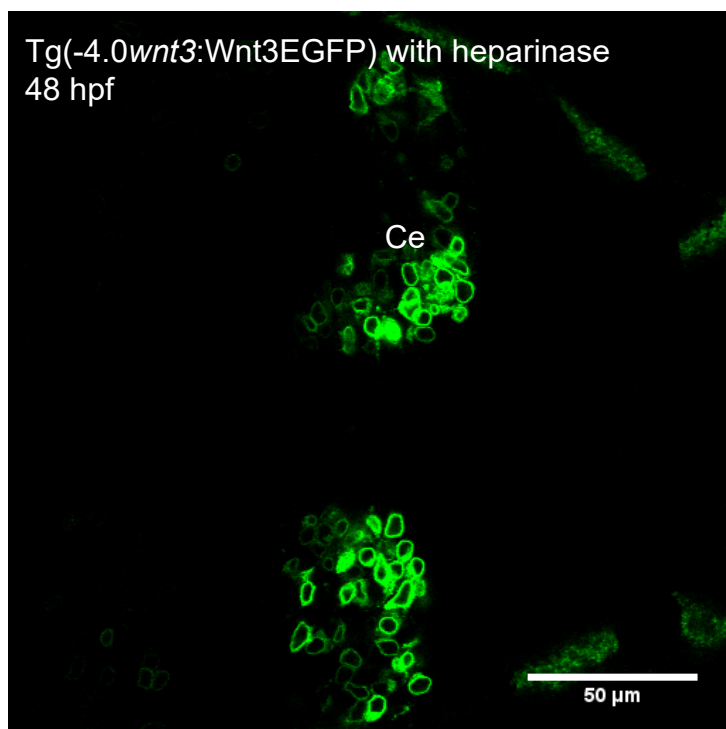**B**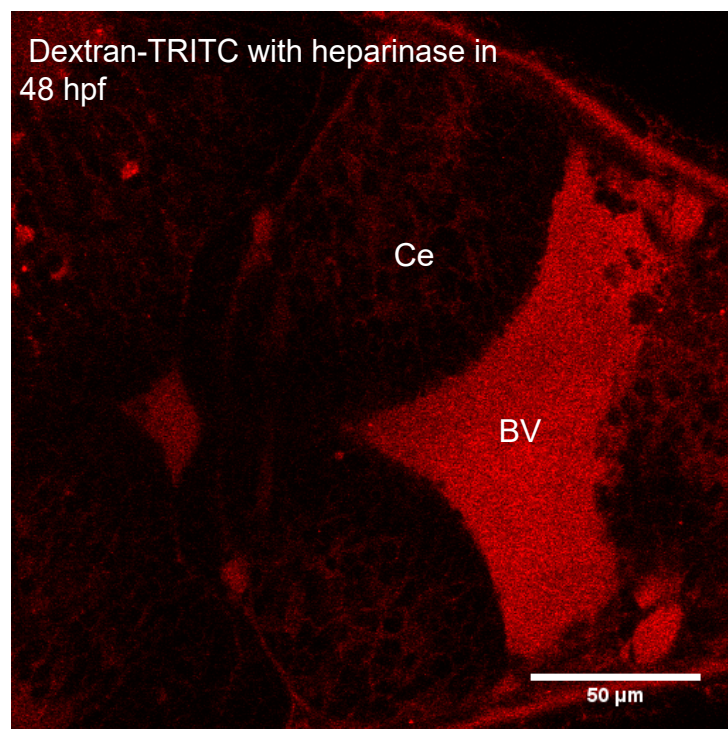**C**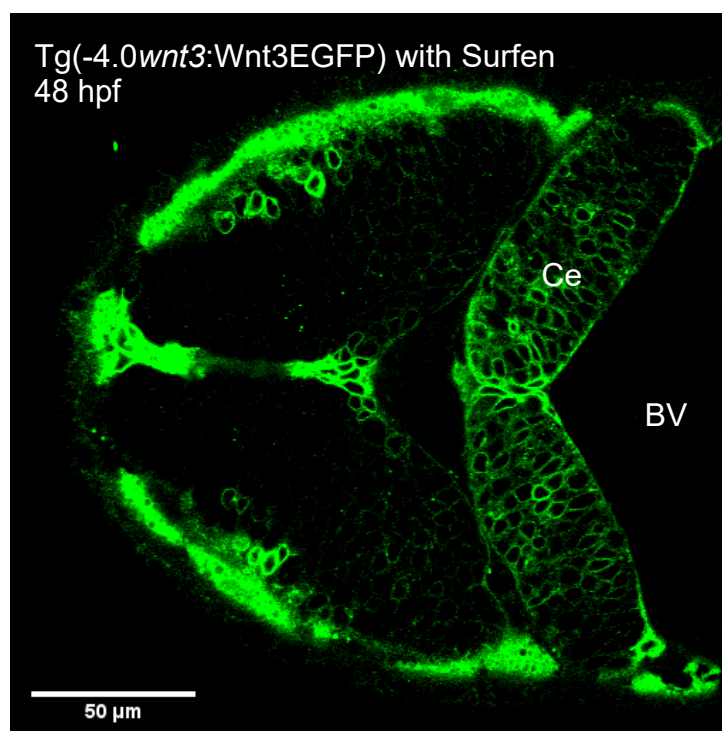

Supplement: Table 1—source data 1. — (A) The expression of Wnt3EGFP after heparinase treatment. (B) Distribution of Dextran-TRITC coinjected with heparinase in the BV of Tg(−4.0wnt3:Wnt3EGFP) embryo. (C) Expression of Wnt3EGFP after surfen treatment. BV, brain ventricle; Ce, cerebellum. Images orientation: anterior to the left. Scale bar 50 μm. [file elife-59489-table1-data1.pdf]
